# Gallstone ileus with an enterovaginal fistula: a rare complication of pelvic radiotherapy

**DOI:** 10.1259/bjrcr.20200060

**Published:** 2020-08-05

**Authors:** Luqman Wali, Fahd Husain, Sharmarke Ali, Sasha Humphries, Linda Turner, Thomas Johnson-Smith, Antony Gough-Palmer

**Affiliations:** 1 Maidstone and Tunbridge Wells NHS Trust, Kent, United Kingdom; 2 Dartford and Gravesham NHS Trust, Kent, United Kingdom; 3 Croydon Health Services NHS Trust, London, United Kingdom

## Abstract

Gallstone ileus is a rare cause of small bowel obstruction. Chronic gallstone irritation can lead to the formation of a cholecystoduodenal fistula, with gallstone impaction typically in the terminal ileum. Rarely gallstones can become impacted in other structures such as the colon or can even erode through the bowel. We present an unusual case of a gallstone ileus which resulted in the formation of an enterovaginal fistula, secondary to previous pelvic radiotherapy. Our case highlights the importance of considering fistula formation as a late complication of radiotherapy and how this can alter expected features of other pathologies, such as a gallstone ileus.

## Clinical presentation

A 76-year-old lady presented to her general practitioner with a 3 week history of diarrhoea and incontinence, after multiple episodes of vomiting. She was treated for FIGO Stage 3C endometrial cancer over 10 years before with a total abdominal hysterectomy, bilateral salpingo-oophorectomy and subsequent chemoradiotherapy. The procedure went well and there were no post-operative complications. However, post-operative histopathology identified positive lymph nodes. Thus, the patient underwent: four cycles of Palitaxel and Carboplatin, 45 Gy in 25 fractions over 5 weeks as external beam radiotherapy, and two insertions of high-dose brachytherapy (8 Gy in two fractions) to the vaginal vault. Following therapy, and after remaining asymptomatic for 5 years, the patient was discharged from routine follow-up. Aside from a recent admission for cholecystitis, she has since remained fit and well with no active medical conditions.

The patient was only able to tolerate liquids and was referred to the emergency department, where she was admitted by the medical team. Initially, the diarrhoea and associated electrolyte dysfunction was thought to be due to pancreatic insufficiency with a superadded urinary tract infection. During further interview, the patient was unsure of the exact origin of the diarrhoea, only that it occurred almost immediately after eating. On further examination, she was found to have sticky yellow discharge from the vagina and excoriation of the legs. A rectovaginal fistula was suspected and she was reviewed by the gynaecology team who recommended urgent pelvic MRI and discussion on the gynaeoncology multidisciplinary team meeting. Unfortunately, there was a slight delay in a formal report being issued. The MRI was initially reviewed by a general radiologist, who reported a pelvic collection and verbally recommended a CT abdomen and pelvis for further evaluation.

## Imaging findings

**Figure 1.**
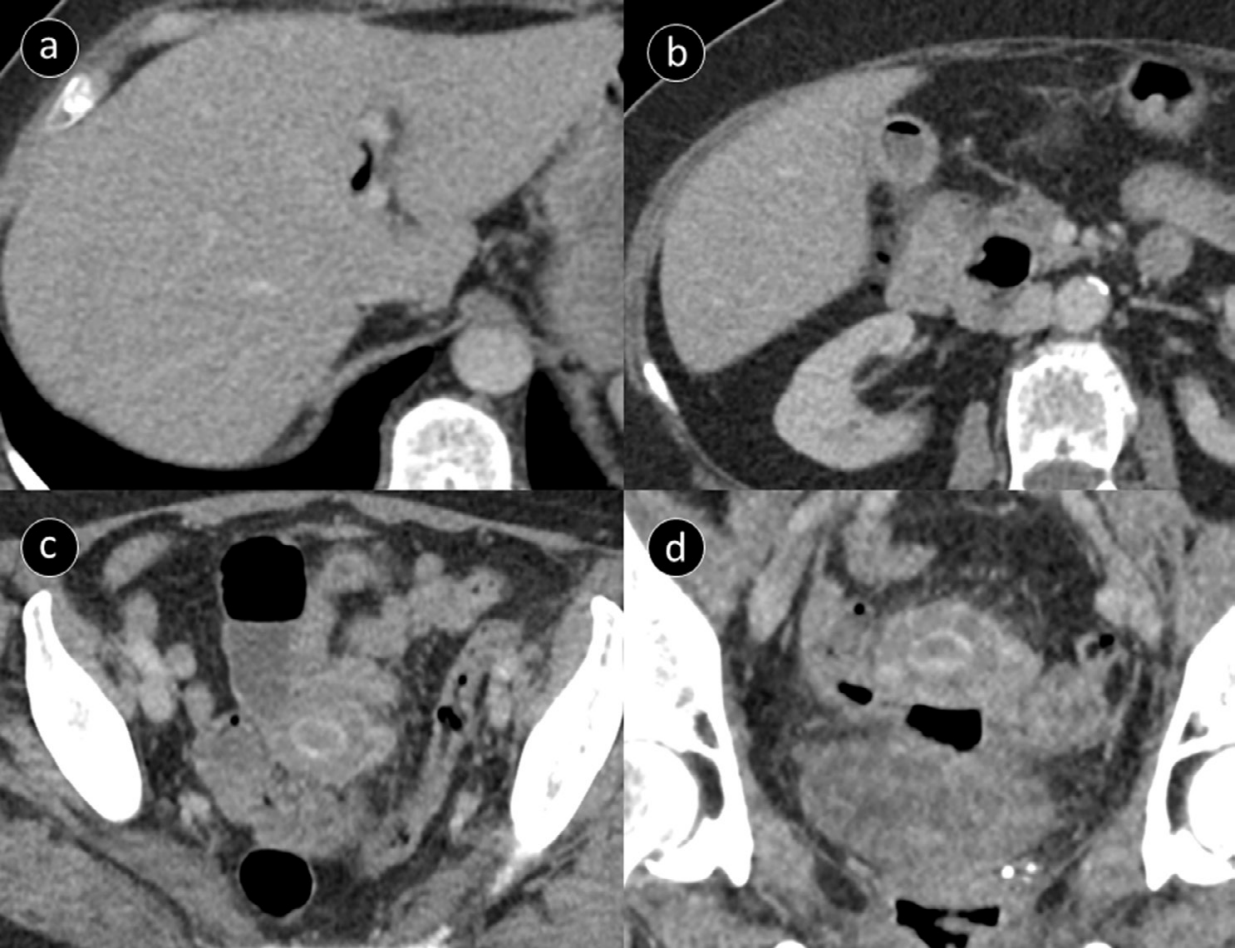
Contrast-enhanced CT performed shortly after admission. (a, b) axial image showing small volume air within the intrahepatic biliary tree and the now empty gallbladder containing air. (c, d) axial and coronal images of the laminated gallstone within a dilated loop of small bowel, note the proximity to the vagina.

**Figure 2.**
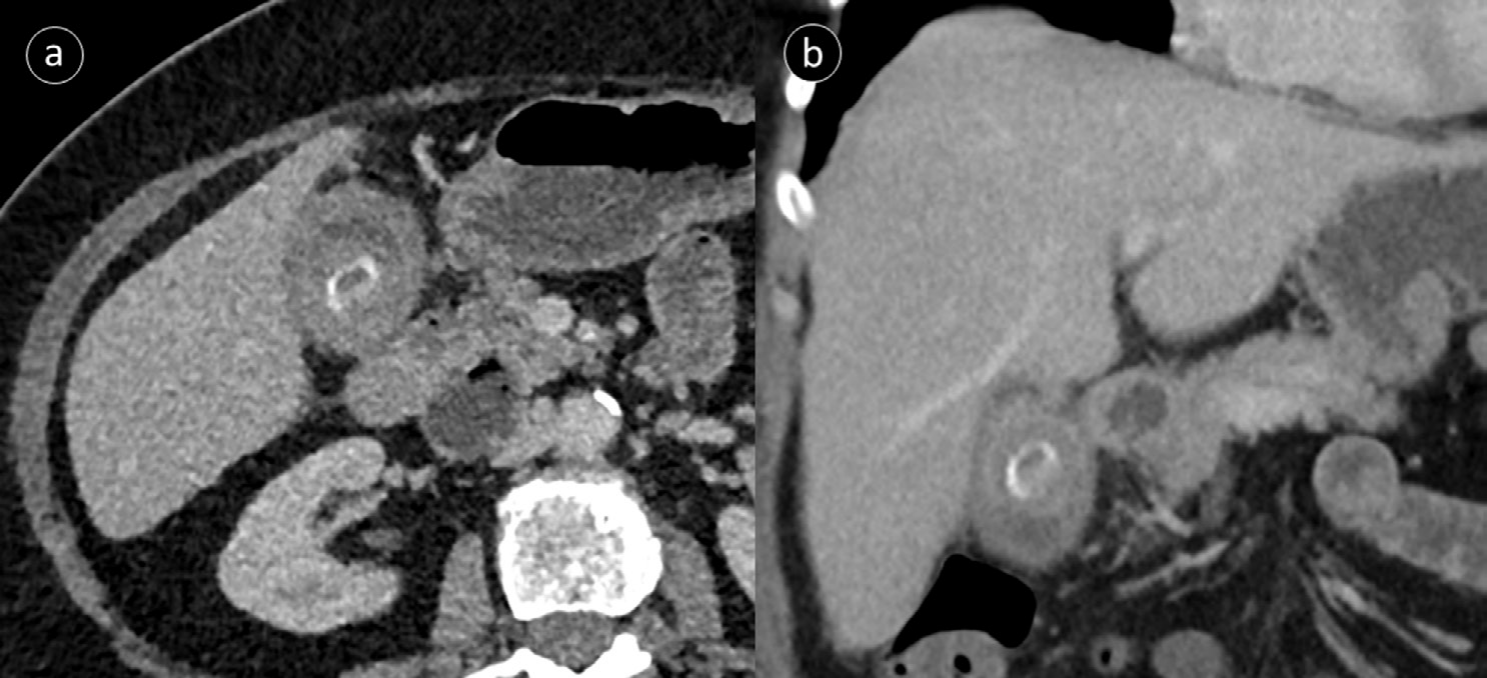
Previous contrast-enhanced CT study performed a year earlier. (a, b) axial and coronal images illustrating the large laminated gallstone located within a contracted gallbladder

A contrast-enhanced CT study of the abdomen and pelvis was performed a few days after the MRI study; although both were reported on the same day. The CT study demonstrated small volume pneumobilia with an empty gallbladder containing air (Figure 1). A laminated hyperdensity was seen within a dilated fluid filled small bowel loop deep within the pelvis, adjacent to the vaginal stump (Figure 1). Mild proximal small bowel dilation was also identified, the distal bowel was of normal calibre. Findings were consistent with a subacute obstruction due to a gallstone ileus. The offending gallstone was seen on a previous CT study, performed during her last admission for acute cholecystitis, which demonstrated a large 2.5 cm gallstone in an inflamed gallbladder (Figure 2).

No definite collection was identified on the more recent CT study although the small bowel loops were inseparable from the vagina. Multiple locules of air were seen around the vagina suggestive of a fistula or localised perforation.

**Figure 3.**
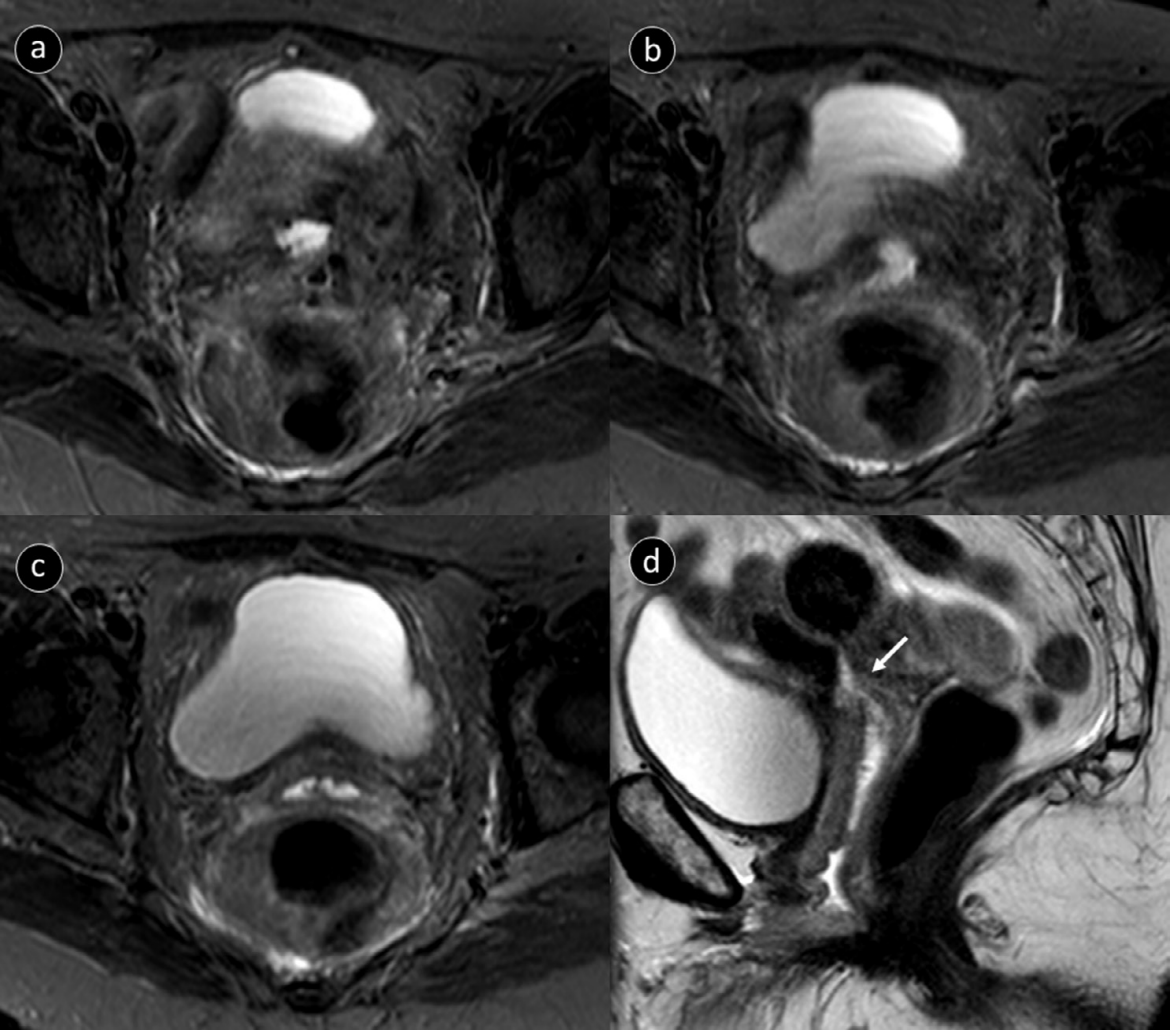
MRI study performed after admission (a, b, c) sequential axial STIR images demonstrating fluid within the small bowel, a fistula containing fluid and fluid within the vagina, respectively. (d) sagittal *T_2_* weighted image demonstrating clearly the fistulous connection (arrow) between the superior vagina and small bowel. STIR, short-tau inversion recovery.

The MRI study confirmed the suspicion of an enterovaginal fistula (Figure 3). This was best seen on STIR and *T*_2_ weighted sequences which demonstrated a focal discontinuity in the superior vagina and adjacent small bowel wall. Fluid signal was seen between the small bowel and the vaginal stump confirming an enterovaginal fistula. There was no evidence of any malignant recurrence on MRI or CT or any other alternative reason for a fistula to form.

## Treatment and outcome

The patient underwent a midline laparotomy, short segment bowel resection and a side-to side-anastomosis. The vaginal discontinuity was closed. The gallstone was found to be impacted slightly distally to the site of perforation with small bowel tethered to the vaginal vault. The patient received routine aftercare in the high dependency unit and surgical ward and was eventually discharged. She reported no further symptoms and is well.

## Discussion

To our knowledge a gallstone ileus causing an enterovaginal fistula is an unreported finding in the literature. Gallstone ileus by itself is a rare cause of small bowel obstruction, the incidence of which increases with age.^1^ Due to its association with elderly patients, who often have multiple co-morbidities or are frail, it has a high mortality. The pathophysiology typically involves a cholecystoduodenal fistula due to chronic inflammation secondary to gallstone formation. The dropped gallstone then lodges in the anatomically narrowest part of the small bowel, most commonly the terminal ileum or ileocaecal valve.^1^ In unusual cases, gallstones can migrate or lodge into the stomach, duodenum or large bowel.^2,3^ Various treatment options are available depending on the size and location of the obstructing stone including enterolithotomy, enterolithotomy and cholecystectomy or open laparotomy with bowel resection.^4^ Minimally invasive enterolithotomy is increasingly becoming more common as it is more suited to this particular cohort of patients, who are high risk candidates for open surgery.

To have superadded radiotherapy complications is extremely rare. In the context of malignancy, fistula formation can be the result of direct involvement of the primary cancer, malignant recurrence or iatrogenic in origin; as a consequence of surgery or radiotherapy.^5^ A well-recognised late complication of radiotherapy is a fistulous connection between the devascularised pelvic organs which can, theoretically, occur between any two structures. More commonly, vesicovaginal and rectovaginal fistulas are encountered; however, enterovaginal and cutaneous involvement can also occur.^5^ Indeed, our patient was already prone to fistula formation, having had pelvic adhesions after completing her treatment. In addition, she was at particularly high risk of having late complications of treatment caused by undergoing external beam radiotherapy and high dose brachytherapy.

We presume that in our patient, the large gallstone had caused the formation of a cholecystoduodenal fistula, much earlier than when she initially presented. Eventually, the gallstone entered the duodenum and became lodged within the distal small bowel, which was likely partially stenosed post-radiotherapy. This is likely to have caused her first episodes of abdominal pain and vomiting. Rather than progressing to a classical gallstone ileus, with complete obstruction, pressure necrosis of the small bowel wall from the gallstone created an enterovaginal fistula. This partially relieved the obstruction with the gallstone eventually becoming impacted more distally. Although gallstone perforation and a solitary case of impaction in a post-radiotherapy sigmoid colon have been reported, no case involving fistulation has previously been identified.^6–8^ This rare case highlights many clinical and radiological lessons.

## Learning points

1. Fistula formation should be considered in patients who have received radiotherapy. MRI is the imaging modality of choice in diagnosing and characterising a fistula.
2. Pelvic radiotherapy can lead to stenosis and stricturing of the bowel.
3. Gallstone ileus is a rare cause of small bowel obstruction and generally increases with age. There is a high associated mortality. Early diagnosis is imperative, where CT is the modality of choice. The common findings include pneumobilia, small bowel obstruction and a focal intraluminal hyperdensity, corresponding to the obstructing gallstone.
4. The degree of obstruction is less severe than expected in the presence of a partially decompressing fistula or perforation.
5. Clinicians should be aware that fistula formation can be a complication of a gallstone ileus.
